# Does co-transplantation of mesenchymal and spermatogonial stem cells improve reproductive efficiency and safety in mice?

**DOI:** 10.1186/s13287-019-1420-9

**Published:** 2019-10-22

**Authors:** Prashant Kadam, Elissavet Ntemou, Jaime Onofre, Dorien Van Saen, Ellen Goossens

**Affiliations:** Biology of the Testis (BITE) Laboratory, Department of Reproduction, Genetics and Regenerative Medicine, Vrije Universiteit Brussel (VUB), Laarbeeklaan 103, 1090 Brussels, Belgium

**Keywords:** Fertility restoration, Male infertility, Mesenchymal stem cells, Spermatogonial stem cells, Transplantation, Reproductive efficiency, Epigenetics

## Abstract

**Background:**

Spermatogonial stem cell transplantation (SSCT) is a promising therapy in restoring the fertility of childhood cancer survivors. However, the low efficiency of SSCT is a significant concern. SSCT could be improved by co-transplanting transforming growth factor beta 1 (TGFβ1)-induced mesenchymal stem cells (MSCs). In this study, we investigated the reproductive efficiency and safety of co-transplanting spermatogonial stem cells (SSCs) and TGFβ1-induced MSCs.

**Methods:**

A mouse model for long-term infertility was used to transplant SSCs (SSCT, *n* = 10) and a combination of SSCs and TGFβ1-treated MSCs (MSi-SSCT, *n* = 10). Both transplanted groups and a fertile control group (*n* = 7) were allowed to mate naturally to check the reproductive efficiency after transplantation. Furthermore, the testes from transplanted males and donor-derived male offspring were analyzed for the epigenetic markers DNA methyltransferase 3A (DNMT3A) and histone 4 lysine 5 acetylation (H4K5ac).

**Results:**

The overall tubular fertility index (TFI) after SSCT (76 ± 12) was similar to that after MSi-SSCT (73 ± 14). However, the donor-derived TFI after MSi-SSCT (26 ± 14) was higher compared to the one after SSCT (9 ± 5; *P* = 0.002), even after injecting half of the number of SSCs in MSi-SSCT. The litter sizes after SSCT (3.7 ± 3.7) and MSi-SSCT (3.7 ± 3.6) were similar but differed significantly with the control group (7.6 ± 1.0; *P* < 0.001). The number of GFP^+^ offspring per litter obtained after SSCT (1.6 ± 0.5) and MSi-SSCT (2.0 ± 1.0) was also similar. The expression of DNMT3A and H4K5ac in germ cells of transplanted males was found to be significantly reduced compared to the control group. However, in donor-derived offspring, DNMT3A and H4K5ac followed the normal pattern.

**Conclusion:**

Co-transplanting SSCs and TGFβ1-treated MSCs results in reproductive efficiency as good as SSCT, even after transplanting half the number of SSCs. Although transplanted males showed lower expression of DNMT3A and H4K5ac in donor-derived germ cells, the expression was restored to normal levels in germ cells of donor-derived offspring. This procedure could become an efficient method to restore fertility in a clinical setup, but more studies are needed to ensure safety in the long term.

## Introduction

Over the last decades, the survival rate after childhood cancer has increased tremendously [1, 2]. However, an important long-term side effect of chemo- and radiotherapy is infertility due to spermatogonial stem cell (SSC) loss. While semen sample storage is not an option for cancer patients diagnosed before puberty, cryostorage of a testicular biopsy prior to the gonadotoxic treatment is highly recommended [3, 4]. For boys facing germ cell loss due to gonadotoxic treatments, transplantation of testicular tissue or SSCs is a promising fertility restoration strategy. In primates, autologous transplantation of pre-pubertal testicular tissue resulted in complete spermatogenesis and the live birth of a healthy monkey [5]. However, for boys having survived a systemic or metastatic cancer, testicular grafting is not an option as contamination of tissue by malignant cells cannot be ruled out [3]. For these boys, SSC transplantation (SSCT) would be the preferred restoration method. SSCT has proved to restore spermatogenesis in various animal models, including non-human primates [6–13]. However, the homing efficiency of SSCs was only 12% [14]. Since chemo- and radiotherapy not only affect SSCs but also the niche cells (Sertoli, Leydig, and peritubular cells) [15, 16], restoring the SSC niche might improve SSCT efficiency.

As mesenchymal stem cells (MSCs) secrete paracrine factors which have anti-apoptotic, anti-inflammatory, and anti-oxidative properties [17–19], they could help to restore the damaged SSC niche [20]. Recently, we showed that transforming growth factor beta 1 (TGFβ1)-induced MSCs improved SSCT efficiency in an infertile mouse model. Although the overall tubular fertility index (TFI) was similar, after co-transplanting TGFβ1-induced MSCs and SSCs (MSi-SSCT), the donor-derived TFI was ten times higher compared to that after SSCT [21]. However, the reproductive efficiency and safety were not evaluated.

The highly complex and dynamic process of epigenetic reprogramming is a crucial step during spermatogenesis. Any abnormality is likely to cause infertility, and the risk of inheriting an altered epigenome resulting in phenotypic defects in offspring cannot be neglected [22, 23].

After SSCT in mice, the expression levels of DNA methyltransferase (DNMT) 1 and 3A and the DNA methylation patterns were not different between controls and first- and second-generation offspring [24]. Moreover, most of the studied stage-specific histone modifications were similar between transplanted males and fertile controls. However, pachytene spermatocytes and round spermatids showed a premature acetylation of lysin 5 on histone 4 after SSCT [25].

To investigate the reproductive efficiency and safety of MSi-SSCT, we performed mating experiments and assessed litter sizes and number of (donor-derived) pups. Furthermore, we evaluated the reproductive safety by detecting the expression pattern of DNMT3A, which is involved in paternal imprinting [26], and histone 4 lysine 5 acetylation (H4K5ac), which plays a role in histone-to-protamine exchange [27], in germ cells of both transplanted males and donor-derived offspring.

## Methods

### Mesenchymal stem cell culture

Red fluorescent protein (RFP)-transfected C57BL/6 mouse bone marrow MSCs (MUBMX-01201; Cyagen Biosciences, Santa Clara, USA) were cultured in T25 cell culture flasks (690,175; Greiner Bio-One, Vilvoorde, Belgium) at a density of 1 × 10^6^ cells/flask with 10 ng/ml recombinant mouse TGFβ1 (P04202; R&D Systems, Minneapolis, USA) in OriCell™ mouse MSC basal medium (MUXMX-90011; Cyagen Biosciences, Santa Clara, USA) in a humidified incubator with 5% CO_2_ at 37 °C for 15–21 days. The medium was changed every third day, and cells were passaged after reaching 80% confluency [21].

### Testicular cell isolation and cryopreservation

Pre-pubertal green fluorescent protein (GFP^+^) F1-hybrid pups (5–7 days), obtained by crossing male inbred C57BL with GFP^+^ female inbred SV129, were used as donors. In these mice, GFP is under control of the β-actin promotor. Testicular cells were isolated from ten donor testes, pooled, and cryopreserved at a concentration of 1 × 10^6^ cells/ml using a slow freezing protocol [28]. At the time of transplantation, the vials were thawed at 37 °C.

### Transplantation experiments

Recipient mice (C57BL/6 J, *n* = 20) were prepared for transplantation by injecting busulfan (40 mg/kg) and cadmium chloride (CdCl_2_, 2 mg/kg) intraperitoneally [21]. Mice were injected twice (1 week before CdCl_2_ injection and 1 week before transplantation) with a subcutaneous dose of the GnRH agonist Decapeptyl (4.26 mg/kg; 0.1 mg; Ipsen, Paris, France) to improve homing and colonization of transplanted cells [29]. Mice were anesthetized by intraperitoneal injection of 75 mg/kg ketamine (Ketamidor®; Ecuphar, Oostkamp, Belgium) and 1 mg/kg medetomidine (Medetor®; Virbac Animal Health, Burgdorf, Germany). A subcutaneous dose (5 mg/kg) of the analgesic meloxicam (Metacam®; Boehringer Ingelheim, Vetmedica GmbH, Ingelheim am Rhein, Germany) was administered preoperatively and for 2 days postoperatively. The surgical area was prepared by clipping the abdominal hair and disinfected with cedium chlorhexidini alcoholicus 0.5% (BE351513; Laboratoires Gifrer Barbezat, Décines-Charpieu, France). The abdomen was incised, and the testes were exteriorized.

Transplantations were performed under a stereomicroscope as previously described [28]. The transplantation was considered “successful” if the tracking dye (trypan blue at the tip of the pipette) along with the injected solution entered the seminiferous tubules. The transplantation was considered “not successful” if the dye and the cell suspension were injected into the interstitium.

**Fig. 1:**
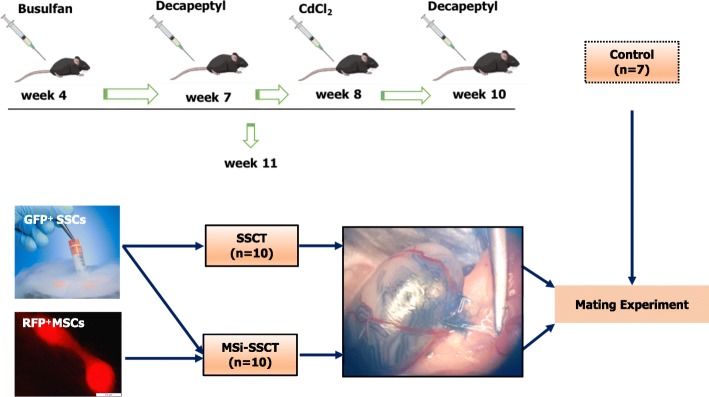
Study design. Transplantation of frozen-thawed GFP^+^ SSCs and/or TGFβ1-treated RFP^+^ MSCs was performed in GFP^−^ mice treated with busulfan and CdCl2. SSCT, spermatogonial stem cell transplantation (*n* = 10); MSi-SSCT, transplantation of TGFβ1-treated MSCs together with SSCs (*n* = 10). Transplanted mice were mated with females 2 months after transplantation. Age-matched fertile mice (*n* = 7) were used as controls for the mating experiment

The experiment consisted of two transplantation groups. For SSCT (*n* = 10), GFP^+^ testicular cells (including SSCs) were resuspended in injection medium [Dulbecco’s modified Eagle’s Medium/F12 (DMEM/F12; Life Technologies, Merelbeke, Belgium) containing 10% penicillin/streptomycin (15140-122; Life Technologies) and 4% fetal calf serum (10500-056; Life Technologies)] to obtain a concentration of 10–20 × 10^6^ cells/ml. For MSi-SSCT (*n* = 10), TGFβ1-treated MSCs at passage 5 or 6 were resuspended in the injection medium to obtain a concentration of 10–20 × 10^6^ cells/ml. GFP^+^ testicular cells (including SSCs) and MSCs were mixed in 1:1 volume. The target was to inject 2 × 10^5^ cells (10 μl) per testis. Non-treated and non-transplanted mice (*n* = 7) were used as fertile controls (Fig. 1).

### Mating experiments

Two months after transplantation, each of the transplanted group (*n* = 9) and control (*n* = 7) mice was housed with two female mice for a period of 3 months. Litter sizes and numbers of GFP^+^ and RFP^+^ pups were recorded. To confirm the origin of the progeny (GFP^+^ SSCs, RFP^+^ MSCs, or endogenous spermatogenesis), the pups were analyzed with a royal blue (excitation 440–460 nm for GFP) and green (excitation 510–540 nm for RFP) led flashlight (Dual Fluorescent Protein Flashlight device; NIGHTSEA, Lexington, USA). All pups were checked for anatomical features, response to external stimuli, and reflexes (righting reflex, holding ability) at 10 and 15 days after birth [30, 31].

### Anatomical and histological analysis

At the end of the mating experiments (5 months after transplantation), recipient males were sacrificed by cervical dislocation. Both transplanted mice and donor-derived offspring were examined thoroughly by a standardized postmortem protocol at anatomical and histological levels [32]. All major organs were collected and checked for abnormalities. Their location and number were noted and critically observed for color, size, shape, consistency, and texture. The testes were collected and decapsulated, fixed in acidified formol alcohol fixative (47608; Sigma-Aldrich, Machelen, Belgium), and embedded in paraffin. The slides were deparaffinized in xylene and rehydrated in a descending series of isopropanol (100%, 100%, 90%, and 70%) followed by a 5-min wash in phosphate-buffered saline (PBS; 70011051; Life Technologies). Endogenous peroxidases were blocked in 0.3% hydrogen peroxide (H3410—500 ml; Sigma-Aldrich) for 30 min. The slides were incubated with 3% normal goat serum for 30 min followed by overnight incubation with the primary mouse anti-GFP antibody (1/200; SC-9996; Santa Cruz, Heidelberg, Germany) at 4 °C. The next morning, the sections were washed three times with PBS for 5 min followed by incubation with a goat anti-rabbit/mouse secondary antibody (K5007; Dako, Heverlee, Belgium) for 1 h at room temperature. After three washes with PBS, 3,3′-diaminobenzidine (1:50; K5007; Dako) was added to visualize the immunoreactivity. The slides were counterstained with hematoxylin. The sections were dehydrated in a mounting series of alcohol (70%, 90%, 100%, and 100%) and xylene. Finally, the slides were mounted using acrytol mounting medium (Surgipath, 10014-986; VWR, Heverlee, Belgium) and analyzed under an Olympus IX 81 inverted bright-field microscope. Adult GFP^+^ and adult GFP^−^ mouse testicular tissue sections with the addition of primary mouse anti-GFP antibody were used as positive and negative controls, respectively. Thirty serial cross-sections per testis (with a 100-μm shift between each slide) were blindly analyzed to assess the overall TFI (percent of tubules containing spermatogenesis) and the donor-derived TFI (percent of tubules containing donor-derived spermatogenesis) [33].

### Analyses of epigenetic markers

The testicular sections were deparaffinized in xylene and rehydrated in a descending series of isopropanol (100%, 100%, 90%, and 70%) followed by a 5-min wash in PBS. Endogenous peroxidases were blocked in 3% hydrogen peroxide for 30 min. For DNMT3A staining, the antigen retrieval step was carried out in a microwave (350 W) with citrate buffer for 10 min, whereas H4K5ac did not require antigen retrieval. For both stainings, non-specific antibody binding was blocked by 5% normal goat serum for 1 h. After each step, the sections were washed in PBS for 5 min. The primary antibodies for DNMT3A (SC-365769; Santa Cruz) and H4K5ac (Ab51997; Abcam, Cambridge, UK) were added at a dilution of 1:250 and 1:1500, respectively, and incubated overnight at 4 °C. For the negative controls, PBS was added instead of the primary antibody. The next day, the sections were washed three times with PBS after which the secondary antibody was added for 1 h at room temperature. The sections were again washed three times with PBS. The visualization of the staining was done with 3,3′-diaminobenzidine (30 s). The sections were immersed in PBS and counterstained with hematoxylin for 1 min. The sections were dehydrated in a mounting series of alcohol (70%, 90% 100%, 100%) and in xylene. Acrytol mounting medium was added before placing the coverslip. For transplanted males, at least ten GFP^+^ tubules per spermatogenic stage were analyzed (Table 1). For controls and offspring, ten serial cross-sections per testis (with a 100-μm shift between each slide) were blindly analyzed to assess the expression of DNMT3A and H4K5ac. For each spermatogenic stage, the localization of the epigenetic marker (cell type) was reported as well as the percentage of round tubules in which the marker was expressed in the respective cell type.

**Table 1:** The 12 stages of the seminiferous epithelial cycle of an adult mouse

	Stages					
	I–IV	V–VI	VII–VIII	IX	X–XI	XII
Level 3	Elongated spt	Elongated spt	Elongated spt			
Level 2	Round spt	Round spt	Round spt	Elongated spt	Elongated spt	Elongated spt
Level 1	Pachytene spc	Pachytene spc	Pachytene spc	Pachytene spc	Pachytene and diplotene spc	2nd meiotic division
Level 0 (basement membrane)	Intermediate spg	B-spg	Preleptotene spc	Leptotene spc	Leptotene and zygotene spc	Zygotene spc

*Spt* spermatid, *spc* spermatocyte, *spg* spermatogonia

### Statistical analyses

(Donor-derived) TFI (mean ± SD) and data of GFP^+^ pups were analyzed by the Mann-Whitney test. TFI in offspring, litter sizes, and the percentage of tubules expressing DNMT3A or H4K5ac were analyzed by one-way ANOVA, followed by Tukey’s post hoc test for multiple comparisons (GraphPad Software version 7, Inc., La Jolla, CA, USA). *P* values < 0.05 are considered statistically significant.

## Results

### Reproductive efficiency

**Fig. 2:**
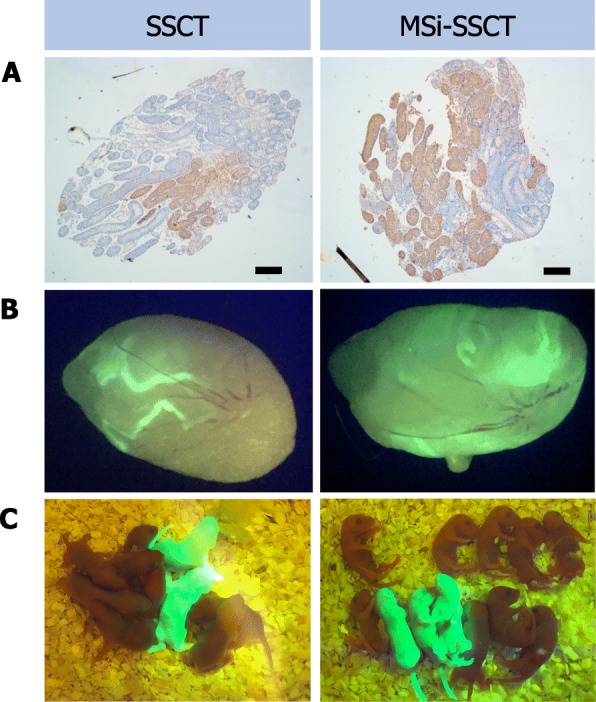
Reproductive efficiency. **a** Five months after SSCT and MSi-SSCT, donor-derived (GFP^+^) spermatogenesis was re-established in the testes of recipients and analyzed by immunohistochemistry. **b** Colonies with donor-derived (GFP^+^) spermatogenesis. **c** Pups obtained after SSCT and MSi-SSCT (brown pups from endogenous spermatogenesis and GFP^+^ pups from donor-derived spermatogenesis). Scale bars = 0.5 mm

SSCT was successful in 85% (17/20) of the testes, while MSi-SSCT was successful in 75% (15/20) of the testes. However, in both transplantation groups, one mouse died after transplantation. These mice were excluded from the analysis. RFP-derived spermatogenesis was never observed. GFP-derived spermatogenesis was found in 47% (7/15) of the successfully injected testes after SSCT and in 62% (8/13) after MSi-SSCT. The overall TFI after SSCT (76 ± 12%) did not differ from the one after MSi-SSCT (73 ± 14%), but the donor-derived TFI after MSi-SSCT (26 ± 14%) was higher compared to the one after SSCT (9 ± 5%; *P* = 0.002). Moreover, TFI in offspring after SSCT (89 ± 3%) and MSi-SSCT (87 ± 4%) was similar to that of control (87 ± 3% (Fig. 2, Table 2). Six out of nine mice (67%) and five out of nine mice (56%) produced offspring after SSCT and MSi-SSCT, respectively. The litter size did not differ between SSCT (3.7 ± 3.7) and MSi-SSCT (3.7 ± 3.6) but differed significantly compared to the control group (7.6 ± 1.0) (Fig. 2, Table 2). However, if only transplanted mice producing offspring were considered, the litter size did not differ from the control, neither after SSCT (7.2 ± 1.1) nor after MSi-SSCT (6.7 ± 1.7). Two mice from the SSCT group and one from the MSi-SSCT group produced GFP^+^ offspring. The number of GFP^+^ offspring per litter did not differ between SSCT (1.6 ± 0.5) and MSi-SSCT (2.0 ± 1.0) (Fig. 2, Table 2). All pups, including GFP^+^ pups, showed normal reflexes and did not show any anatomical abnormalities. No unusual anatomical findings were encountered during the gross postmortem examination in the major visceral (heart, liver, lungs, kidneys, and spleen) and reproductive organs. Their location and number were found to be normal as well as the color, size, shape, consistency, and texture. No anomalies were detected.

**Table 2:** Reproductive efficiency

	Control	SSCT	MSi-SSCT
No. of males*	7	9	9
No. of males with offspring	7	6	5
No. of males with donor-derived offspring (GFP^+^)	0	2	1
TFI	_	76 ± 12%	73 ± 14%
Donor-derived TFI	_	9 ± 5%	26 ± 14%^a^
TFI in offspring	87 ± 3%	89 ± 3%	87 ± 4%
Litter size	7.6 ± 1.0	3.7 ± 3.7^b^	3.7 ± 3.6^b^
Litter size (only mice with offspring)	7.6 ± 1.0	7.2 ± 1.1	6.7 ± 1.7
Number of GFP^+^ offspring per litter	_	1.6 ± 0.5	2.0 ± 1.0

^*^Each male mouse was housed with two female mice for a period of 3 months

^a^*P* = 0.002 compared to SSCT

^b^*P* < 0.001 compared to control

### Reproductive safety

#### Epigenetic markers in germ cells of transplanted males

**Fig. 3:**
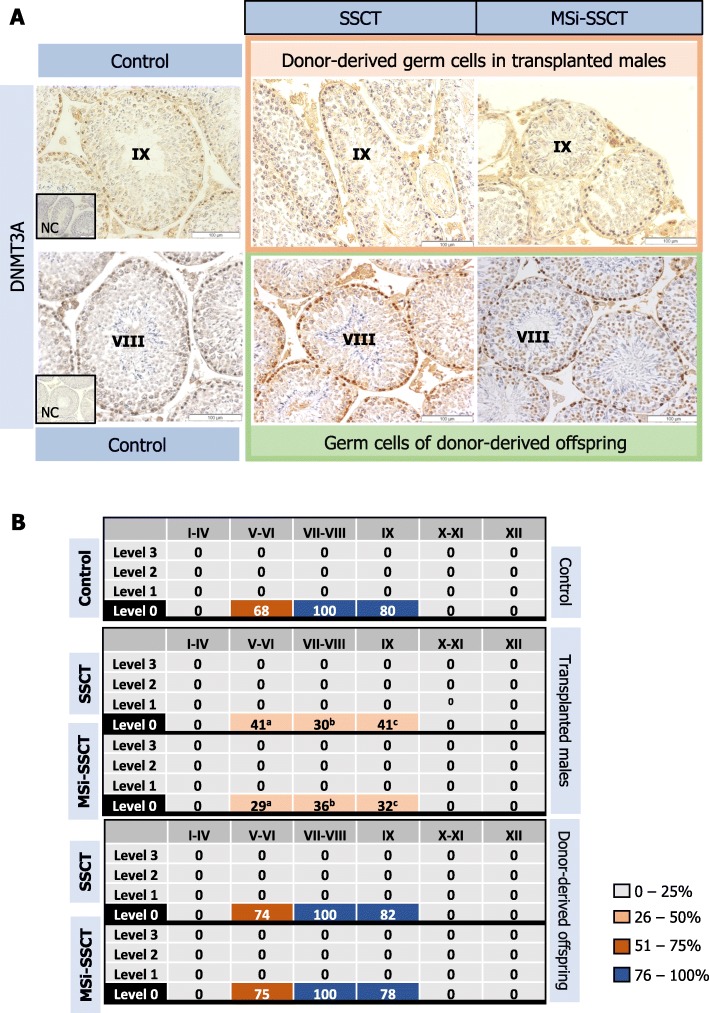
Expression of DNMT3A. **a** DNMT3A expression in control germ cells, donor-derived germ cells in transplanted males, and germ cells of donor-derived offspring obtained after transplantation. **b** For each spermatogenic stage, the percentage of tubules expressing the marker was determined (^a^*P* < 0.005; ^b,c^*P* < 0.001 compared to control)

**Fig. 4:**
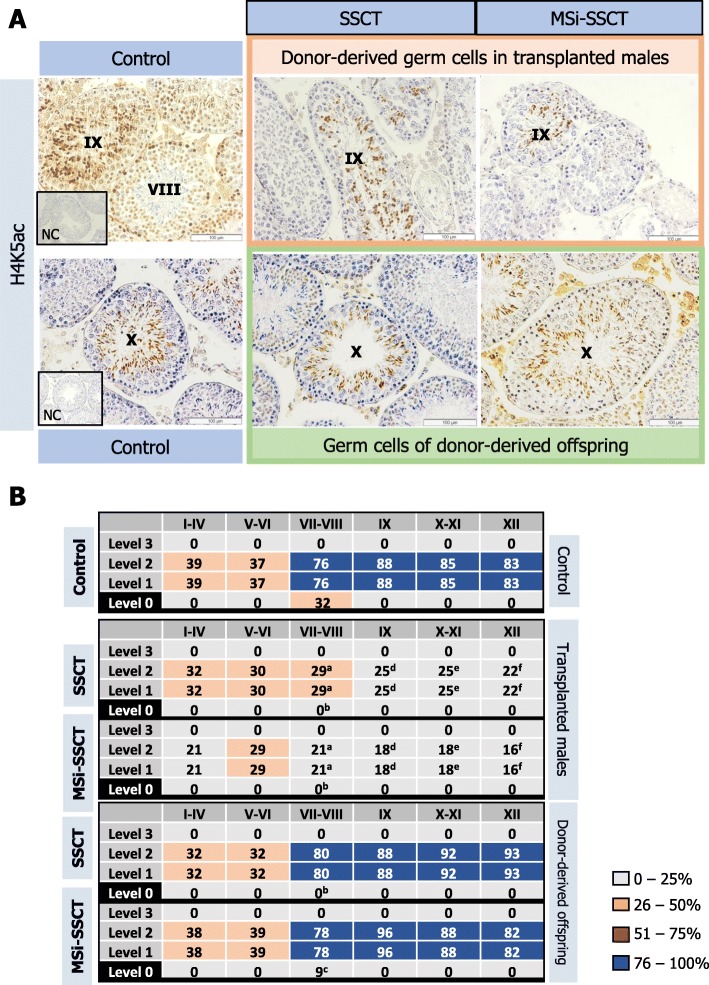
Expression of H4K5ac. **a** H4K5ac in control germ cells, donor-derived germ cells in transplanted males, and germ cells of donor-derived offspring obtained after transplantation. **b** For each spermatogenic stage, the percentage of tubules expressing the marker was determined (^a,b,d,e,f^*P* < 0.001; ^c^*P* < 0.005 compared to control)

Tubules containing donor-derived spermatogenesis were evaluated for DNMT3A (Fig. 3a) and H4K5ac expression (Fig. 4a). DNMT3A expression was only detected in spermatogonia and (pre)leptotene spermatocytes in tubules in stages V–IX (Fig. 3b). Although the staining pattern was similar to controls, DNMT3A expression was significantly reduced in both transplanted groups (stages V–VI: *P* < 0.005; stages VII–VIII and IX: *P* < 0.001). H4K5ac was detected in spermatocytes and round spermatids but not in elongated spermatids. Interestingly, pre-leptotene spermatocytes also showed H4K5ac in the control group but not in any of the transplanted groups. Overall, H4K5ac showed a similar pattern in transplanted males and control mice, but in transplanted males, the expression was lower from stage VII onwards (*P* < 0.001) (Fig. 4b).

#### Epigenetic markers in germ cells of donor-derived offspring

DNMT3A was expressed in spermatogonia and (pre)leptotene spermatocytes. The expression pattern was similar between control and donor-derived offspring (Fig. 3). H4K5ac was detected in spermatocytes and round spermatids but not in elongated spermatids (Fig. 4). Overall, H4K5ac showed a similar pattern in the donor-derived offspring and controls. Only pre-leptotene spermatocytes in stages VII–VIII show reduced acetylation.

## Discussion

Previously, we showed that the co-transplantation of MSCs improved the efficiency of SSCT. However, before MSi-SSCT can become a clinical application, the efficiency and safety need to be confirmed as well. Although MSCs did not trans-differentiate towards germ cells, their paracrine factors might have played a role in restoring the damaged SSC niche and thus improving the efficiency of transplantation [21]. Whether this improved efficiency translates into a better reproductive efficiency, and safety was the subject of the present study. After natural conception, the reproductive efficiency of MSi-SSCT was similar to that of SSCT, but the donor-derived TFI significantly higher after MSi-SSCT. This finding was consistent with our previous report [21]. However, it has to be noted that in MSi-SSCT, only half of the number of SSCs were transplanted in relation to SSCT. This has a significant impact on a future clinical application considering the low numbers of SSCs that can be obtained from pre-pubertal testicular biopsies. From our previous study, we learned that, compared to MSCs, TGFβ1-treated MSCs showed significantly lower expression of IL6, MCP1, MMP3, TCK1, KC, and MIP1G which have been previously reported to play a role in inflammation and migration [34–37]. Inhibition of these paracrine factors by TGFβ1 might have reduced the migratory property of MSCs retaining them in the testis. This might have contributed to SSC homing resulting in improved colonization and proliferation [21].

RFP^+^ MSC-derived pups were not observed. Indeed, also in our previous study, RFP^+^ MSC-derived spermatogenesis could not be observed, although few MSCs co-expressed the germ cell marker MVH [21].

Although TFI was similar for SSCT and MSi-SSCT, the donor-derived TFI after MSi-SSCT was higher than that after SSCT. Moreover, the donor-derived TFI after MSi-SSCT accounted for 36% of the total TFI whereas it only accounted for 12% after SSCT (*P* = 0.002). This difference was not seen in the proportions of GFP^+^ pups. Thirty percent (2.0/6.7) of the pups were GFP^+^ after MSi-SSCT, and 22% (1.6/7.2) were GFP^+^ after SSCT (*P* > 0.05). However, it has to be mentioned that only two SSCT and one MSi-SSCT transplanted male produced GFP^+^ pups. This could be due to the fact that donor-derived spermatogenesis was in competition with spontaneously recovered or MSC-induced endogenous spermatogenesis. If an SSC niche is occupied with an endogenous SSC, it cannot harbor a transplanted SSC, as the niche can only contain one SSC [38]. The reduced DNMT3A and H4K5ac expression in the donor-derived germ cells could have attributed as well. However, offspring obtained after SSCT as well as MSi-SSCT showed similar TFI and DNMT3A and H4K5ac expression as that of fertile control.

Proper epigenetic modifications are crucial for the germ cells as they are responsible for the (epi)genetic inheritance to the next generations [22, 23]. The male germ cell-specific epigenetic imprinting is acquired prenatally in diploid gonocytes followed by further consolidation after birth during spermatogenesis [23] which implies the importance of having two checkpoints: one after transplantation and one in the offspring. Therefore, we evaluated the reproductive safety by assessing the stage-specific expression levels of DNMT3A and H4K5ac in germ cells of transplanted males and donor-derived offspring. DNMT3A expression was only detected in spermatogonia and (pre)leptotene spermatocytes which is consistent with the findings from Watanabe et al. [39], La Salle and Trasler [40], and Goossens et al. [25]. In transplanted mice, but not in the offspring, the expression level of DNMT3A and H4K5ac was significantly lower compared to controls. The damaged testicular microenvironment might be responsible for the reduced levels of DNMT3A and H4K5ac expression in donor-derived germ cells. As a similar pattern of DNMT3A and H4K5ac expression was seen after SSCT and MSi-SSCT, the paracrine signaling from TGFβ1-treated MSCs did not affect the epigenetic marks.

Aberrations in sperm DNA methylation are correlated with poor sperm quality and impaired fertility [41]. Moreover, H4K5ac is also one of the critical factors of post-translational modifications (histone-to-protamine exchange), which play a crucial role in spermatogenesis and sperm function [42]. Thus, the reduced DNMT3A and H4K5ac expression in donor-derived germ cells might have resulted in incompetent spermatozoa, and this might explain the low numbers of GFP^+^ pups.

Although we reported H4K5ac up to leptotene spermatocytes before [25], we only observed H4K5ac in preleptotene spermatocytes in the control and MSi-SSCT groups but not in the SSCT group. This difference could be due to the fact that we used cryopreserved SSCs whereas fresh SSCs were used in the previous study. Cryopreservation has been shown to cause epigenetic aberrations in DNA methylation and histone modification in various cell types, including germ cells [43]. In addition, we used a different model (busulfan/cadmium-treated mice vs. genetically infertile W/W^v^ mice). In contrast to the present model, somatic cells from W/W^v^ mice have never supported spermatogenesis, which may have interfered with epigenetic modification. SSCT using fresh SSCs resulted in significantly higher donor-derived TFI (35%), compared to using cryopreserved SSCs, which resulted only in a donor-derived TFI of only 9% (*P* < 0.001) [44]. This confirms the negative impact of the cryopreservation process. Transplanted SSCs have to transmigrate through the blood-testis barrier in order to find their niche in the seminiferous tubules. This phenomenon is called “homing” [45]. Harsh cryopreservation conditions (enzymatic digestion, freezing, and thawing) might damage the cell wall due to the packing effect [46], which might impact SSC homing efficiency. As there is a growing interest in epigenetics and trans-generational effects [47], it would be interesting to further investigate whether cryopreservation protocols and SSC homing influence transgenerational epigenetics.

Although our data look promising for a future clinical application, we have to be aware that much more epigenetic marks are being reprogrammed during gametogenesis and that the method used in this study is semi-quantitative. For the exact quantification of epigenetic modifications, recent improvements in single-cell analytics are promising. Epigenetic mechanisms can now be explored in-depth using advanced techniques (e.g., BS-seq and TAB-seq for gene-specific DNA methylation, ChIP-seq for histone modifications), even on single-cell level (for review [48]). Moreover, these techniques could distinguish heterogeneous cell populations within a tissue and their epigenome fluctuations [49].

Although MSCs offer an enormous regenerative potential, they also pose some difficulties for clinical implementation such as their diverse sources, individual variability, and lack of standardized protocols for in vitro maintenance and differentiation [50]. These difficulties could be bypassed using exosomes to a certain extent. Exosomes (40–100-nm membrane vesicles) are of endocytic origin and are released by many cells in vitro containing various kinds of RNAs and secretory proteins [51, 52]. Exosomes from MSCs could play a role in tissue regeneration and homeostasis. Future experiments could investigate whether MSC-derived exosomes also play a supportive role.

It would have been interesting to evaluate the transplantation of TGFβ-treated MSCs alone. In our previous study [21], we included the transplantation of non-treated MSCs and showed that MSCs could help to restore endogenous spermatogenesis. Also, in the group transplanted with both SSCs and TGFβ-treated MSCs, endogenous spermatogenesis recovered. Including a group transplanted with TGFβ-treated MSCs alone could have solidified our results and conclusions.

Another limitation of the study is that testicular cell suspensions (including immature germ cells, Sertoli and Leydig cells) were transplanted instead of isolated SSCs. Although we acknowledge that SSC sorting could have improved SSCT efficiency [53]. Parreira et al. [54] showed that most of the transplanted cells are flushed away or eliminated from the seminiferous epithelium by Sertoli cells through phagocytosis by the end of the first week after transplantation. Only SSCs are able to home in the niche and further multiply and colonize within the seminiferous tubules. Moreover, specific markers to identify functional SSCs have not been reported [55]. The golden standard to detect functional SSCs is transplanting the cells to sterilized mice. Therefore, the proportion of SSCs can vary between repeated procedures. We tried to reduce this variability by using pooled samples from ten donor testes.

Moreover, in a future clinical application, xeno-derived products, like fetal calf serum, need to be replaced by human-derived alternatives (e.g., human serum albumin).

## Conclusion

Co-transplanting SSCs and TGFβ1-treated MSCs reach the reproductive potential of SSCT alone even after transplanting half the number of SSCs. Although low expression of DNMT3A and H4K5ac were observed in donor-derived gem cells, normal levels were restored in offspring. However, extensive epigenetic analyses are needed in order to ensure reproductive safety.

## Data Availability

The datasets used and/or analyzed during the current study are available from the corresponding author on request.

## Abbreviations

CdCl_2_: Cadmium chloride
DMEM/F12: Dulbecco’s modified Eagle’s medium/F12
DNMT3A: DNA methyltransferase 3A
GFP: Green fluorescent protein
H4K5ac: Histone 4 lysine 5 acetylation
MSCs: Mesenchymal stem cells
MSi-SSCT: A combination of SSCs and TGFβ1-treated MSCs
PBS: Phosphate-buffered saline
RFP: Red fluorescent protein
SD: Standard deviation
SSCs: Spermatogonial stem cells
SSCT: Spermatogonial stem cell transplantation
TFI: Tubular fertility index
TGFβ1: Transforming growth factor β1
